# Risk factors for linezolid-associated hyponatremia focused on differences between intravenous and oral administration: a single-center, retrospective study

**DOI:** 10.1186/s40780-025-00463-0

**Published:** 2025-06-20

**Authors:** Ryoji Takata, Masatoshi Taga, Hirofumi Nagai, Yoshihiro Nishita, Hironori Kobayashi, Nozomi Arakawa, Toru Imai, Yoshitsugu Iinuma, Togen Masauji

**Affiliations:** 1 Department of Pharmacy, Kanazawa Medical University Hospital, 1-1 Daigaku, Uchinada, Kahoku, Ishikawa 920-0293 Japan; 2 Department of Pharmacy, Nihon University Itabashi Hospital, 30-1 Ohyaguchi-Kami-Cho, Itabashi-Ku, Tokyo, 173-8610 Japan; 3 Department of Infectious Disease, Kanazawa Medical University, 1-1 Daigaku, Uchinada, Kahoku, Ishikawa 920-0293 Japan

**Keywords:** Linezolid, Hyponatremia, Administration route, Propensity score matching

## Abstract

**Background:**

Linezolid (LZD)-associated hyponatremia is a rare side effect, and no reports have compared intravenous and oral administration in relation to the development of hyponatremia. This study aimed to identify risk factors for LZD-associated hyponatremia and to evaluate whether there are differences in the development of hyponatremia between intravenous and oral administration.

**Methods:**

We conducted a retrospective study that included patients aged ≥ 20 years who received LZD of 1200 mg/day intravenously or orally at Kanazawa Medical University Hospital from January 2011 to December 2023. Patient information was retrospectively examined, and multiple logistic regression analysis was used to assess the risk of intravenous administration for hyponatremia. Additionally, propensity scores were calculated for the intravenous and oral administration groups, and these scores were subsequently used in a propensity score matching analysis.

**Results:**

This retrospective study revealed hyponatremia in 32 of 240 (13.3%) patients. Intravenous administration (OR = 17.137, 95% CI = 2.029–144.712, *P* = 0.009), serum sodium level before administration (OR = 0.626, 95% CI = 0.528–0.744, *P* < 0.001), and creatinine clearance (OR = 0.987, 95% CI = 0.975–0.999, *P* = 0.040) were identified as independent variables associated with hyponatremia. After propensity score matching, the incidence of LZD-associated hyponatremia was higher with intravenous administration than with oral administration (OR = 9.697, 95% CI = 1.153–81.545,* P* = 0.029).

**Conclusions:**

This study identified intravenous administration as an independent risk factor for LZD-associated hyponatremia, and that the risk of hyponatremia was significantly higher with the intravenous administration compared with the oral administration. Patients with the identified risk factors should be administered intravenous LZD more cautiously and carefully monitored for serum sodium levels.

## Background

Linezolid (LZD) demonstrates approximately 100% bioavailability and is administered both intravenously and orally to treat infections targeting gram-positive pathogens. Recent case reports have indicated hyponatremia as a potential adverse effect of LZD [1–4].

Hyponatremia is one of the major electrolyte abnormalities observed in clinical practice [5], and it is associated with prolonged hospitalization and increased mortality [6]. Hyponatremia is categorized into hypotonic, isotonic, and hypertonic. Most clinically observed hyponatremia is hypotonic and is induced by drugs, syndrome of inappropriate antidiuretic hormone secretion (SIADH), heart failure, renal failure, massive water intake, and insufficient sodium intake [5, 7, 8].

Risk factors for LZD-associated hyponatremia include high C-reactive protein (CRP) [9], concomitant potassium-sparing diuretics administration [9], advanced age [10], low baseline serum sodium levels [10], and low serum albumin levels [11, 12]. However, no reports have compared intravenous and oral administration in relation to the development of hyponatremia. Sodium-free solution (5% dextrose solution) of 300 mL is administered simultaneously with intravenous LZD; thus, a daily free water intake of 600 mL may induce hypotonic hyponatremia. Therefore, we hypothesized that there is a difference in the incidence of hyponatremia between intravenous and oral administration of LZD. This study aimed to verify our research hypothesis and assess the risk factors for LZD-associated hyponatremia.

## Methods

This study is a single-center retrospective study. The Ethical Review Committee of Kanazawa Medical University Hospital approved the study before initiation (approval number: H355), and opt-outs were implemented. Informed consent was waived by the committee because of the retrospective study design. This study was conducted under the Declaration of Helsinki and described following the Strengthening the Reporting of Observational Studies in Epidemiology statement.

### Participants

This study included patients aged ≥ 20 years who received LZD of 1200 mg/day (twice daily) intravenously or orally from January 2011 to December 2023 at Kanazawa Medical University Hospital. Patients with continuous hemofiltration, hemodialysis, or peritoneal dialysis, with a SIADH diagnosis, and with a pre-administration serum sodium level (Pre-Na: serum sodium level up to 2 days before LZD administration) of < 130 mmol/L were excluded, as well as those with serum sodium levels that were not measured at three points: baseline sodium level (Baseline-Na: serum sodium level from 4 to 21 days before LZD administration), Pre-Na, and lowest sodium level (Nadir-Na: lowest serum sodium level during the LZD administration). Additionally, patients whose Pre-Na dropped by ≥ 10 mmol/L from Baseline-Na and those in whom the duration between the end of intravenous administration and the start of oral administration was < 5 days were excluded. Typically, a 3% saline solution is used to treat hyponatremia. The administration of 500 mL of a 3% saline solution provides approximately 250 mmol of sodium. Therefore, patients who received more than 250 mmol/day of sodium, including that obtained from intravenous infusions and diet, were also excluded.

### Data collection and hyponatremia definition

Patient data were retrospectively collected from electronic medical records. The observation period was until the end of LZD administration. Patient information, including sex, age, weight and medical history, and laboratory data, such as hemoglobin, white blood cell count, platelet count, CRP, serum sodium, serum potassium, serum chloride, serum albumin, serum creatinine, aspartate aminotransferase, and alanine aminotransferase, were collected. The Cockcroft–Gault formula was used to calculate creatinine clearance (CCr) [13]. Three points of serum sodium levels were collected: Baseline-Na, Pre-Na, and Nadir-Na. It was difficult to investigate the in–out balance of the patients, therefore, the volume of administered infusion fluid, amount of infusion sodium intake, and level of dietary sodium intake were investigated to the extent possible. The infusion fluid volume and the sodium amount in the infusion fluid were calculated from all infusions and solutions administered on the day prior to the Nadir-Na measurement, which was considered to have the greatest impact on Nadir-Na. Dietary sodium intake was calculated from the amount of salt in each meal and the percentage of each meal consumed on the day prior to the Nadir-Na measurement. Concomitant medications that could potentially contribute to hyponatremia were investigated, including loop diuretics, thiazides, potassium-sparing diuretics, vasopressin, antidepressants/antipsychotics, and anticancer drugs (vinca alkaloids, platinum compounds, and alkylating agents) [14]. Anticancer drugs were included as concomitant medications despite their single dose rather than daily administration. Tolvaptan was included as a concomitant medication because it can cause hypernatremia. Concomitant antibacterial and antifungal agents were also collected. Data on infection focus were obtained from medical records and classified into 10 categories: sepsis, catheter-related infection, febrile neutropenia, pneumonia, osteomyelitis or infectious arthritis, infectious meningitis or brain abscess, skin and soft tissue infection, urinary tract infection, others, and unknown.

Hyponatremia was defined as grade ≥ 2, that is Nadir-Na of < 130 mmol/L, as assessed by the Common Terminology Criteria for Adverse Events version 5.0 [15].

### Statistical analysis

JMP version 17.2.0 software (SAS Institute, Cary, NC, USA) was used for all statistical analysis. Data were presented as numerical values (%) for categorical variables and medians (interquartile range) for continuous variables. All analyses were two-tailed, with *P*-values of < 0.05 considered statistically significant.

#### Risk factors for LZD-associated hyponatremia

All patients were classified into hyponatremic and non-hyponatremic groups. Patient’s characteristics were analyzed using Fisher’s exact test for categorical variables and Mann–Whitney *U* test for continuous variables. Hyponatremia-associated risk factors were identified by univariate and multiple logistic regression analyses. The objective variable was the development of hyponatremia. As explanatory variables were the factors with *P*-values of < 0.100 in univariate analysis. However, factors of concern for multicollinearity were excluded. These factors were entered into a multiple logistic regression model, and variable selection was performed with a stepwise method based on *P*-values.

#### Propensity score matching

All patients were classified into intravenous and oral groups and a logistic regression analysis model was used to calculate propensity scores. The model incorporated the following pre-dose patient background factors as covariates: sex, age, weight, infection focus, medical history, hemoglobin, white blood cell count, platelet count, CRP, Pre-Na, serum potassium, serum chloride, serum albumin, serum creatinine, CCr, aspartate aminotransferase, alanine aminotransferase and concomitant medications. One-to-one matching was performed using propensity scores to adjust for differences in patient background characteristics between the intravenous and oral administration groups to the greatest extent possible. Propensity score matching was performed using a caliper width of 0.1. After propensity score matching, Fisher’s exact test was performed, with the onset of hyponatremia as the objective variable and intravenous administration as the explanatory variable.

## Results

### Patients and incidence of hyponatremia

**Fig. 1:**
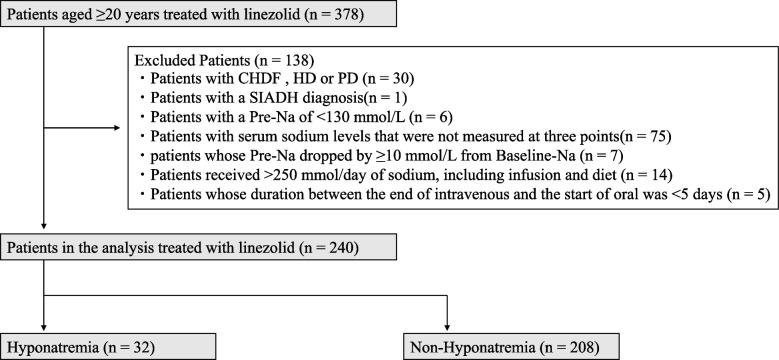
Flowchart of patient selection following the inclusion criteria of this study. The flowchart illustrates the number of patients enrolled in the study and included in the analysis, as well as the number of excluded, along with the reasons for exclusion. *CHDF*: continuous hemodiafiltration, *HD*: hemodialysis, *PD*: peritoneal dialysis, *SIADH*: syndrome of inappropriate secretion of antidiuretic hormone

This study included 378 patients. Of them, 138 who met the exclusion criteria were excluded. Finally, 240 patients met the inclusion criteria, of whom 32 (13.3%) developed hyponatremia (Fig. 1). The median decrease in serum sodium concentration was 8 mmol/L in the hyponatremia group and 2 mmol/L in the non-hyponatremia group. The median total dosing duration was 10 days (interquartile range: 7–15 days).

### Risk factors for LZD-associated hyponatremia

Table 1 presents the patient characteristics of the hyponatremia and non-hyponatremia groups. Among the factors with *P*-values of < 0.100 in the univariate analysis, Baseline-Na was excluded due to multicollinearity with Pre-Na, kidney failure was excluded due to multicollinearity with CCr, and sodium intake from infusions and solutions was excluded due to multicollinearity with infusion fluid volume. Therefore, the following factors were incorporated into a multiple logistic regression model, and factors were selected using a stepwise method: intravenous, age, Pre-Na, white blood cell count, serum potassium, serum chloride, aspartate aminotransferase, CCr, serum albumin, sodium intake from dietary, infusion fluid volume, loop diuretics. The result indicates that the intravenous administration (odds ratios (OR) = 17.137, 95% confidence interval (CI) = 2.029–144.712, *P* = 0.009), Pre-Na (OR = 0.626, 95% CI = 0.528–0.744, *P* < 0.001), and CCr (OR = 0.987, 95% CI = 0.975–0.999, *P* = 0.040) were independent risk factors for hyponatremia (Table 2).

**Table 1:** Patient characteristics in the hyponatremia and non-hyponatremia groups

Characteristics	Hyponatremia (*n* = 32)	Non-Hyponatremia (*n* = 208)	*P-*Value
Intravenous/Oral; *n*	31 (96.9)/1 (3.1)	153 (73.6)/55 (26.4)	0.003 b
Sex (male/female); *n* (%)	21 (65.6)/11 (34.4)	136 (65.4)/72 (34.6)	1.000 b
Age (years)	79 [73–83]	74 [64–80]	0.010 a
Body weight (kg)	51.1 [44.6–61.8]	54.5 [46.9–62.9]	0.145 a
Days to Nadir-Na (day)	6.5 [4.8–9.0]	ND	-
Baseline-Na (mmol/L)	136 [133–138]	140 [137–142]	< 0.001 a
Pre-Na (mmol/L)	133 [132–136]	139 [136–142]	< 0.001 a
Nadir-Na (mmol/L)	127 [125–129]	137 [134–139]	< 0.001 a
Decreased serum sodium (mmol/L)	–8 [–10 – –6]	–2 [–4–0]	< 0.001 a
Hemoglobin (g/dL)	8.9 [7.9–9.8]	9.0 [7.6–10.6]	0.545 a
White blood cell count (× 10^3^/μL)	7.84 [4.47–11.63]	5.75 [1.27–10.39]	0.071 a
Platelet count (× 10^5^/μL)	143 [34–277]	170 [34–300]	0.980 a
Serum potassium (mmol/L)	4.1 [3.8–4.5]	3.9 [3.4–4.3]	0.096 a
Serum chloride (mmol/L)	99 [96–102]	103 [101–106]	< 0.001 a
Aspartate aminotransferase (IU/L)	32 [17–57]	24 [16–37]	0.046 a
Alanine aminotransferase (IU/L)	32 [15–66]	24 [15–44]	0.233 a
Serum creatinine (mg/dL)	0.84 [0.61–1.25]	0.76 [0.60–1.21]	0.452 a
Creatinine clearance (mL/min)	49.6 [33.1–74.4]	58.7 [36.0–93.3]	0.061 a
C-reactive protein (mg/dL)	8.1 [5.3–14.0]	6.1 [2.7–13.8]	0.149 a
Serum albumin (g/dL)	2.2 [1.8–2.7]	2.6 [2.1–3.1]	0.011 a
Sodium intake from infusions and solutions (mmol/day)	96 [45–151]	46 [0–95]	< 0.001 a
Sodium intake from dietary (mmol/day)	16 [0–52]	65 [0–103]	0.003 a
Infusion fluid volume (mL/day)	1795 [1044–2150]	1100 [600–1900]	0.002 a
Total sodium intake (mg/day)	135 [74–198]	116 [89–149]	0.289 a
Basal disease; *n* (%)			
Cancer	15 (46.9)	116 (55.8)	0.446 b
Diabetes	9 (28.1)	52 (25.0)	0.669 b
Kidney failure	11 (34.4)	36 (17.3)	0.031 b
Heart failure	4 (12.5)	27 (13.0)	1.000 b
Ischemic heart disease	5 (15.6)	26 (12.5)	0.578 b
Infection; n (%)			
Sepsis	2 (6.3)	4 (1.9)	0.183 b
Catheter-related infection	3 (9.4)	19 (9.1)	1.000 b
Febrile neutropenia	2 (6.3)	29 (13.9)	0.393 b
Pneumonia	10 (31.3)	73 (35.1)	0.842 b
Osteomyelitis or infectious arthritis	4 (12.5)	20 (9.6)	0.538 b
Infectious meningitis or brain abscess	0 (0)	7 (3.4)	0.598 b
Skin and soft tissue infection	5 (15.6)	25 (12.0)	0.567 b
Urinary tract infection	2 (6.3)	11 (5.3)	0.686 b
Others	4 (12.5)	15 (7.2)	0.295 b
Unknown	0 (0)	5 (2.4)	1.000 b
Concomitant drugs; *n* (%)			
β-Lactamase inhibitors/Penicillin	2 (6.3)	32 (15.4)	0.273 b
Carbapenems	14 (43.8)	69 (33.2)	0.318 b
Trimethoprim-sulfamethoxazole	4 (12.5)	25 (12.0)	1.000 b
Antifungal drug	10 (31.3)	66 (46.5)	1.000 b
Loop diuretics	14 (43.8)	44 (21.2)	0.013 b
Thiazide diuretics	1 (3.1)	1 (0.5)	0.249 b
Potassium-sparing diuretics	2 (6.3)	14 (6.7)	1.000 b
Vasopressin	0 (0)	0 (0)	-
Antipsychotic	4 (12.5)	34 (16.3)	0.795 b
Anticancer drugs	0 (0)	0 (0)	-
Tolvaptan	1 (3.1)	6 (2.9)	1.000 b

aMann–Whitney *U* test for continuous data (median (interquartile range))

bFisher’s exact test for categorical data (%)

**Table 2:** Multivariate analysis of demographic and clinical data in the hyponatremia and non-hyponatremia

	OR	95% CI	*P-*value
Intravenous	17.137	2.029–144.712	0.009
Pre-Na	0.626	0.528–0.744	< 0.001
CCr	0.987	0.975–0.999	0.040

*OR* odds ratio, *CI* confidence interval, *Pre-Na* pre-administration serum sodium level, *CCr* creatinine clearance

### Propensity score matching

Table 3 shows the characteristics of patients in the intravenous and oral administration groups.

**Table 3:** Patient characteristics in the intravenous and oral groups

Characteristics	Intravenous (*n* = 184)	Oral (*n* = 56)	*P-*Value
Sex (male/female); *n* (%)	122 (66.3)/62 (33.7)	35 (62.5)/21 (37.5)	0.632 b
Age (years)	74 [64–81]	74 [67–80]	0.739 a
Body weight (kg)	54.2 [47.0–63.0]	52.3 [45.9–60.0]	0.389 a
Pre-Na (mmol/L)	138 [135–141]	139 [136–141]	0.483 a
Hemoglobin (g/dL)	8.8 [7.6–10.2]	9.8 [8.3–10.8]	0.036 a
White blood cell count (× 10^3^/μL)	6.04 [1.37–10.65]	5.34 [3.10–9.19]	0.474 a
Platelet count (× 10^5^/μL)	146 [34–290]	220 [83–332]	0.141 a
Serum potassium (mmol/L)	3.9 [3.5–4.3]	4.1 [3.5–4.4]	0.208 a
Serum chloride (mmol/L)	103 [100–106]	103 [101–106]	0.911 a
Aspartate aminotransferase (IU/L)	25 [16–39]	22 [15–31]	0.061 a
Alanine aminotransferase (IU/L)	28 [17–52]	19 [11–31]	0.004 a
Serum creatinine (mg/dL)	0.76 [0.59–1.19]	0.83 [0.64–1.29]	0.240 a
Creatinine clearance (mL/min)	59.4 [35.8–90.2]	51.9 [35.9–80.3]	0.271 a
C-reactive protein (mg/dL)	7.4 [3.8–14.5]	5.2 [1.2–8.0]	0.003 a
Serum albumin (g/dL)	2.5 [2.1–3.1]	2.8 [2.3–3.1]	0.040 a
Basal disease; *n* (%)			
Cancer	109 (59.2)	22 (39.3)	0.010 b
Diabetes	47 (25.5)	14 (25.0)	1.000 b
Kidney failure	35 (19.0)	12 (21.4)	0.703 b
Heart failure	22 (12.0)	9 (16.1)	0.494 b
Ischemic heart disease	17 (9.2)	14 (25.0)	0.005 b
Infection; n (%)			
Sepsis	6 (3.3)	0 (0)	0.341 b
Catheter-related infection	19 (10.3)	3 (5.4)	0.426 b
Febrile neutropenia	26 (14.1)	5 (8.9)	0.370 b
Pneumonia	69 (37.5)	14 (25.0)	0.108 b
Osteomyelitis or infectious arthritis	14 (7.6)	10 (17.9)	0.039 b
Infectious meningitis or brain abscess	6 (3.3)	1 (1.8)	1.000 b
Skin and soft tissue infection	19 (10.3)	11 (19.6)	0.103 b
Urinary tract infection	7 (3.8)	6 (10.7)	0.083 b
Others	14 (7.6)	5 (8.9)	0.779 b
Unknown	4 (2.2)	1 (1.8)	1.000 b
Concomitant drugs; *n* (%)			
β-Lactamase inhibitors/Penicillin	24 (13.0)	10 (17.9)	0.384 b
Carbapenems	73 (39.7)	10 (17.9)	0.002 b
Trimethoprim-sulfamethoxazole	27 (14.7)	2 (3.6)	0.033 b
Antifungal drug	66 (35.9)	10 (17.9)	0.013 b
Loop diuretics	46 (25.0)	12 (21.4)	0.722 b
Thiazide diuretics	1 (0.5)	1 (1.8)	0.413 b
Potassium-sparing diuretics	9 (4.9)	7 (12.5)	0.063 b
Antipsychotic	32 (17.4)	6 (10.7)	0.298 b
Tolvaptan	5 (2.7)	2 (3.6)	0.666 b

aMann–Whitney *U* test for continuous data (median (interquartile range))

bFisher’s exact test for categorical data (%)

Propensity score matching was performed to adjust for patient background factors, resulting in 41 matched patients in each group for subsequent analyses. Table 4 presents the characteristics of patients in the intravenous and oral administration groups after matching. Further analysis revealed that intravenous administration was associated with a significantly higher risk of hyponatremia compared to oral administration (OR = 9.697, 95% CI = 1.153–81.545,* P* = 0.029) (Table 5).

**Table 4:** Patient characteristics in the intravenous and oral groups after propensity score matching

Characteristics	Intravenous (*n* = 41)	Oral (*n* = 41)	SMD
Sex (male/female); *n* (%)	27 (65.9)/14 (34.1)	27 (65.9)/14 (34.1)	< 0.001
Age (years)	72 [64–80]	74 [67–79]	0.039
Body weight (kg)	55.0 [46.9–61.1]	53.4 [46.4–61.1]	0.103
Pre-Na (mmol/L)	139 [135–141]	139 [136–142]	0.135
Hemoglobin (g/dL)	9.5 [7.7–11.5]	9.7 [8.3–10.7]	0.004
White blood cell count (× 10^3^/μL)	5.39 [2.43–7.24]	5.55 [1.00–10.14]	0.164
Platelet count (× 10^5^/μL)	220 [53–335]	215 [23–339]	0.049
Serum potassium (mmol/L)	3.9 [3.5–4.2]	4.1 [3.5–4.4]	0.093
Serum chloride (mmol/L)	103 [100–106]	103 [101–106]	0.020
Aspartate aminotransferase (IU/L)	20 [15–51]	20 [14–33]	0.223
Alanine aminotransferase (IU/L)	24 [15–40]	20 [12–43]	0.022
Serum creatinine (mg/dL)	0.83 [0.62–1.22]	0.78 [0.61–1.00]	0.035
Creatinine clearance (mL/min)	54.3 [34.7–83.0]	54.5 [41.7–96.1]	0.176
C-reactive protein (mg/dL)	5.7 [2.4–11.1]	5.4 [1.5–9.5]	0.044
Serum albumin (g/dL)	2.7 [2.3–3.4]	2.7 [2.2–3.1]	0.167
Basal disease; *n* (%)			
Cancer	19 (46.3)	19 (46.3)	< 0.001
Diabetes	12 (29.3)	10 (24.4)	0.110
Kidney failure	7 (17.1)	6 (14.6)	0.067
Heart failure	5 (12.2)	4 (9.8)	0.078
Ischemic heart disease	7 (17.1)	9 (22.0)	0.123
Infection; n (%)			
Sepsis	0 (0)	0 (0)	-
Catheter-related infection	3 (7.3)	3 (7.3)	< 0.001
Febrile neutropenia	5 (12.2)	4 (9.8)	0.078
Pneumonia	10 (24.4)	12 (29.3)	0.110
Osteomyelitis or infectious arthritis	7 (17.1)	5 (12.2)	0.138
Infectious meningitis or brain abscess	2 (4.9)	1 (2.4)	0.130
Skin and soft tissue infection	6 (14.6)	8 (19.5)	0.130
Urinary tract infection	3 (7.3)	4 (9.8)	0.087
Others	4 (9.8)	4 (9.8)	< 0.001
Unknown	1 (2.4)	0 (0)	0.224
Concomitant drugs; *n* (%)			
β-Lactamase inhibitors/Penicillin	6 (14.6)	8 (19.5)	0.130
Carbapenems	6 (14.6)	9 (22.0)	0.190
Trimethoprim-sulfamethoxazole	2 (4.9)	2 (4.9)	< 0.001
Antifungal drug	9 (22.0)	8 (19.5)	0.060
Loop diuretics	9 (22.0)	6 (14.6)	0.190
Thiazide diuretics	1 (2.4)	1 (2.4)	< 0.001
Potassium-sparing diuretics	5 (12.2)	5 (12.2)	< 0.001
Antipsychotic	4 (9.8)	5 (12.2)	0.078
Tolvaptan	2 (4.9)	1 (2.4)	0.130

continuous data (median (interquartile range))

categorical data (%)

*SMD* standardized mean difference

**Table 5:** Comparison of incidence of hyponatremia between intravenous and oral groups after propensity score matching

	Intravenous (*n* = 41)	Oral (*n* = 41)	OR	95% CI	*P-*value
Hyponatremia	8	1	9.697	1.153–81.545	0.029
Non-Hyponatremia	33	40			

*OR* odds ratio, *CI* confidence interval

*P-*Values, odds ratio, and 95%CI were obtained by Fisher’s exact test comparing Injection group and Oral group after propensity score matching

## Discussion

In the present retrospective study, we found that intravenous administration was a risk factor for LZD-associated hyponatremia and that intravenous administration was associated with a higher risk of hyponatremia compared with oral administration. The incidence of hyponatremia in this study was 13.3% (32/240), which was higher than the 7% in a phase III clinical trial conducted in Japan [16]. However, the rate of LZD-associated hyponatremia has been relatively higher in previous reports [9–12, 17], and a similar trend was found in the present study.

The pharmaceutical differences between intravenous and oral LZD administration include blood concentration and free water administration. Regarding blood concentration, LZD achieved almost equivalent blood concentrations both intravenously and orally [18]; thus, hyponatremia may be independent of LZD blood concentration. Hyponatremia is primarily a water balance disorder caused by a relative excess of water compared with the total sodium content of the body [5]. Although sodium intake (solute) is also affected, the contribution of the extracellular fluid volume (solvent) is larger. Therefore, dilution of body fluids by 600 mL of free water per day during intravenous administration could explain the difference in hyponatremia incidences between the intravenous and oral administration. However, the mechanism of LZD-associated hyponatremia is unclear. Future studies to elucidate the mechanism of hyponatremia should be conducted on each administration route because the present results indicate that the differences among the intravenous and oral administration affect the hyponatremia incidence. Additionally, developing a powder vial formulation in which the dissolving solution can be changed following a patient’s condition is desirable.

Pre-Na and CCr were detected as other risk factors for LZD-associated hyponatremia. Pre-Na was identified as a risk factor, possibly because this study defined hyponatremia using absolute values of < 130 mmol/L, rather than a decreasing rate. Several studies reported that patients with chronic kidney disease are prone to hyponatremia [19, 20], a finding consistent with the present study. Patients with these risk factors, in addition to intravenous administration, may be at higher risk of LZD-associated hyponatremia, indicating that patients with multiple risk factors should be monitored more carefully for serum sodium levels. In this retrospective study, the patient who developed hyponatremia due to oral administration had a Pre-Na of 134 mmol/L and a CCr of 40.6 mL/min, both of which had risk factors identified in this study.

The result interpretation of this study has several limitations. First, as a single-center retrospective study, the influence of potential confounding factors cannot be entirely excluded. Vomiting might have contributed to the development of hyponatremia [21]; however, its impact could not be fully assessed due to the missing data in this retrospective study. In the study cohort, 12.5% (4/32) of the patients who developed hyponatremia experienced vomiting during the administration period and only one patient received concomitant antiemetic therapy, suggesting that the severity of vomiting was mild and might not have significantly contributed to the development of hyponatremia. Second, we were unable to evaluate indices such as Sequential Organ Failure Assessment score or Acute Physiologic Assessment and Chronic Health Evaluation II score that assess the severity of infection. Severe infections are known to increase vasopressin secretion by producing inflammatory cytokines, including tumor necrosis factor-α and interleukin-6 [22]. Although, inflammation-related indicators, such as CRP and white blood cell count, and infection focus were not identified as independent risk factors for LZD-associated hyponatremia in this study, the severity of infection would play a role in developing hyponatremia. Third, the infusion volume was studied, but we were unable to assess the actual total fluid intake, including the volume of water taken by the patients. Similarly, sodium intake from sources other than meals, such as supplements and beverages, could not be evaluated. However, it is difficult to rigorously assess water and sodium intake from these in retrospective studies. Prospective studies with adjustment for patient background factors are required to prove that the mechanism of LZD-associated hyponatremia includes dilutional hyponatremia due to free water administration.

## Conclusions

In the present study, we found that intravenous administration of LZD was associated with LZD-associated hyponatremia and that this route of administration was associated with a higher risk of hyponatremia compared with oral administration. This is the first report that compared LZD-associated hyponatremia, focusing on the differences between intravenous and oral administration. Intravenous LZD should be used with greater caution, and serum sodium levels should be carefully monitored in patients with risk factors.

## Data Availability

No datasets were generated or analysed during the current study.

## Abbreviations

LZD: Linezolid
SIADH: Syndrome of inappropriate secretion of antidiuretic hormone
CRP: C-reactive protein
OR: Odds ratio
CI: Confidence interval
